# A mixed-methods evaluation of the MORE^OB^ program in Ontario hospitals: participant knowledge, organizational culture, and experiences

**DOI:** 10.1186/s12913-019-4224-9

**Published:** 2019-07-08

**Authors:** Jessica Reszel, Deborah Weiss, Ann E. Sprague, Deshayne B. Fell, Sandra Dunn, Mark C. Walker, Dana Sidney, Monica Taljaard, Wendy E. Peterson

**Affiliations:** 1 Better Outcomes Registry & Network (BORN) Ontario, 401 Smyth Road, Ottawa, Ontario K1H 8L1 Canada; 2 Children’s Hospital of Eastern Ontario (CHEO) Research Institute, 401 Smyth Road, Ottawa, Ontario K1H 8L1 Canada; 3 School of Epidemiology and Public Health, University of Ottawa , 600 Peter Morand Crescent, Ottawa, Ontario K1G 5Z3 Canada; 4 Clinical Epidemiology, Ottawa Hospital Research Institute (OHRI), 501 Smyth Road, Ottawa, Ontario K1H 8L6 Canada; 5 Department of Obstetrics and Gynecology, University of Ottawa , 501 Smyth Road, Ottawa, Ontario K1H 8L6 Canada; 6 School of Nursing, University of Ottawa, 451 Smyth Road, Ottawa, Ontario K1H 8M5 Canada

**Keywords:** Obstetrics and gynecology, Patient safety, Qualitative research, Safety culture, Surveys

## Abstract

**Background:**

MORE^OB^ (Managing Obstetrical Risk Efficiently) is a patient safety program for health care providers and administrators in hospital obstetric units. MORE^OB^ has been implemented widely in Canada and gradually spread to the United States. The main goal of MORE^OB^ is to build a patient safety culture and improve clinical outcomes. In 2013, 26 Ontario hospitals voluntarily accepted provincial funding to participate in MORE^OB^. The purpose of our study was to assess the effect of MORE^OB^ on participant knowledge, organizational culture, and experiences implementing and participating in the program at these 26 Ontario hospitals.

**Methods:**

A convergent parallel mixed-methods study in Ontario, Canada, with MORE^OB^ participants from 26 hospitals. The quantitative component used a descriptive pre-post repeated measures design to assess participant knowledge and perception of culture, administered pre-MORE^OB^ and after each of the three MORE^OB^ modules. Changes in mean scores were assessed using mixed-effects regression. The qualitative component used a qualitative descriptive design with individual semi-structured interviews. We used content analysis to code, categorize, and thematically describe data. A convergent parallel design was used to triangulate findings from data sources.

**Results:**

308 participants completed the knowledge test, and 329 completed the culture assessment at all four time points. Between baseline and post-Module 3, statistically significant increases on both scores were observed, with an increase of 7.9% (95% CI: 7.1 to 8.8) on the knowledge test and an increase of 0.45 (on a scale of 1–5, 95% CI: 0.38 to 0.52) on the culture assessment. Interview participants (*n* = 15) described improvements in knowledge, interprofessional communication, ability to provide safe care, and confidence in skills. Facilitators and barriers to program implementation and sustainability were identified.

**Conclusions:**

Participants were satisfied with their participation in the MORE^OB^ program and perceived that it increased health care provider knowledge and confidence, improved safety for patients, and improved communication between team members. Additionally, mean scores on knowledge tests for obstetric content and culture assessment improved. The MORE^OB^ program can help organizations and individuals improve care by concentrating on the human and organizational aspects of patient safety. Further work to improve program implementation and sustainability is required.

**Electronic supplementary material:**

The online version of this article (10.1186/s12913-019-4224-9) contains supplementary material, which is available to authorized users.

## Background

There are over 380,000 births in Canada each year [1] and the majority occur in hospitals [2]. Improvements in women’s health and health care, as well as advancements in prenatal and obstetric care have led to decreases in maternal and neonatal mortality rates [3]. However, recent trends in maternal characteristics, including older age of mothers and higher rates of obesity, have resulted in increased rates of some maternal and neonatal adverse events [4]. In 1999, the Institute of Medicine’s landmark report *To Err Is Human* identified adverse events, many of which are preventable, as a common cause of morbidity and mortality in hospitals and called for a coordinated strategy to improve patient safety [5]. Although the report set a goal of reducing adverse events by 50% over five years, there remains room for improvement. For example, in Canada in 2014–2015, 1 in 18 hospital stays (or 138,000 hospitalizations) involved at least one occurrence of potentially preventable harm [6]. While obstetric and newborn patients have a lower rate of harm compared to other patient groups (with a harm rate of 4.2 per 100 patients, and 1.0 per 100 patients, respectively) [6], obstetrical departments are often a focus for patient safety and quality improvement. There is a need to prioritize patient safety in obstetrics due to the unique challenges of working in labour and delivery [7], the fact that an adverse outcome may affect more than one patient, and due to the high cost of liability insurance and claims [8, 9], with obstetrics accounting for nearly half of the Healthcare Insurance Reciprocal of Canada’s (HIROC) liability claims and patient compensation payments [10].

The MORE^OB^ (Managing Obstetrical Risk Efficiently) program was developed by the Society of Obstetricians and Gynaecologists of Canada (SOGC), and is currently administered by Salus Global, a healthcare consulting and implementation firm established by the SOGC and HIROC in 2007 [11]. The MORE^OB^ program aims to create a culture of patient safety in obstetrical units by using high reliability organization (HRO) principles [12],including awareness of systems that influence patient care and outcomes, focusing on near-misses as opportunities to improve processes, a culture that promotes open communication and teamwork, and a commitment to ongoing training and learning [13]. The program contains three evidence-informed modules: Learning Together, Working Together, and Changing the Culture [14]. The program requires that all obstetric team members at enrolled hospitals (e.g., nurses, midwives, family physicians, obstetricians, administrators) jointly participate in the three modules, each of which is implemented over approximately one year. MORE^OB^ uses a “train-the-trainer” approach whereby the hospital recruits a “core team” of interdisciplinary team members, who are then trained and supported by Salus Global to implement the program at their respective hospitals. Standardization of implementation across sites is facilitated by detailed program manuals and implementation responsibilities for the core team, orientation sessions by MORE^OB^ facilitators prior to implementation of each module, and ongoing support by program consultants [14]. Additional information about the MORE^OB^ program goals is provided in Additional file 1.

To date, over 300 North American hospitals have participated in the MORE^OB^ program [12], but there are limited studies and variable outcomes. In Canada, the province of Alberta implemented the MORE^OB^ program in all hospitals in 2004, and a subsequent evaluation found statistically significant reductions in perineal tears, length of stay, and neonatal severe morbidity rates [15]. No significant changes were observed for postpartum infection, postpartum hemorrhage with caesarean section, or fetal mortality [15]. One Ontario study reported that participation in the MORE^OB^ program resulted in a significant decrease in the number of reportable events and associated costs for the healthcare liability insurer [16]. Another Ontario study found that participation in the MORE^OB^ program did not translate to an observable decrease in adverse maternal or neonatal birth outcomes [17]. However, data collected from Canadian sites showed increases in participant knowledge and organizational safety culture [18].

In 2002 in the province of Ontario, hospitals providing obstetrical services began to voluntarily implement the program, and by 2012, 67 hospitals were involved with MORE^OB^. In 2013, the Ontario Ministry of Health and Long-Term Care (MOHLTC) offered to fund the program for the remaining 34 provincial hospitals that were not yet engaged with MORE^OB^, and 26 hospitals accepted and completed the program. The implementation of the MORE^OB^ program at these Ontario hospitals provided an opportunity to conduct a mixed-methods evaluation of the program implementation. This evaluation aimed to assess the outcomes and local implementation process for the MORE^OB^ program in the Ontario context, provide data to facilitate comparison between provincial settings, and contribute qualitative data for more in-depth information on participant experiences with the program.

## Methods

**Fig. 1:**
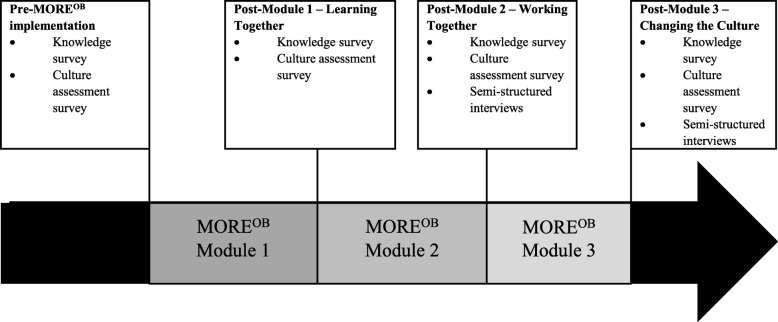
Quantitative and qualitative data sources

This study used a convergent parallel mixed-methods design [19, 20]. Simultaneous data collection and subsequent analysis and integration of data from quantitative (i.e., surveys) and qualitative (i.e., semi-structured interviews) sources allowed us to compare and corroborate our findings from different data sources (Fig. 1). Our research team was not involved in implementing the MORE^OB^ program in any of the sites but conducted the comprehensive evaluation of the program with two main goals, specifically to evaluate (1) the program outcomes and (2) the implementation process (Table 1).

**Table 1:** Overall evaluation of MORE^OB^ program in Ontario

		Objective		
Evaluation of MORE^OB^ program in Ontario	Evaluation of outcomes	Objective 1	For the cohort of 67 Ontario hospitals where the MORE^OB^ program was implemented between 2002 and 2012:	To quantitatively evaluate the effect of the MORE^OB^ program on maternal and neonatal outcomes (reported in [17]).
		Objective 2a	For the cohort of 26 Ontario hospitals newly enrolled in the MORE^OB^ program in 2013:	To quantitatively and qualitatively evaluate the effect of the MORE^OB^ program on participants’ knowledge about obstetrical core content contained in the MORE^OB^ program.
		Objective 2b		To quantitatively and qualitatively evaluate the effect of the MORE^OB^ program on participants’ perceptions of their organizational culture.
	Evaluation of process	Objective 2c		To qualitatively evaluate barriers and facilitators to implementing and participating in the MORE^OB^ program.

This paper reports the methods and results for objectives 2a, 2b, and 2c only. The outcomes of knowledge and culture were selected as they were aligned with the MORE^OB^ program goals (Additional file 1), and the data available from existing surveys administered routinely as part of participation in the MORE^OB^ program. The evaluation of the implementation process aimed to identify barriers and facilitators to implementing and participating in the program to provide insight into why the program may or may not have worked in different settings and to identify areas for improvement.

### Surveys

#### Design

We used a descriptive pre-post repeated measures design to assess the effect of the MORE^OB^ program on participants’ knowledge about obstetrical core content contained in the MORE^OB^ program and to assess the effect of the MORE^OB^ program on participants’ perceptions of their organizational culture.

#### Measures

We used data from two existing surveys administered routinely to all participants by Salus Global as part of involvement in the MORE^OB^ program:

1. An online, standardized, 75-item multiple-choice knowledge test previously developed by the MORE^OB^ program which assesses core clinical knowledge covered in the program. The knowledge test had a maximum score of 93, however for ease of interpretation, we transformed the score to a percentage (D Walker, 2015, unpublished; [18]).
2. A previously developed and validated online 54-item Culture Assessment Survey (CAS) [21] to assess participants’ perceptions of the culture of patient safety in their organization. As designed, the CAS is divided into six categories (patient safety is everyone’s priority, teamwork, valuing individuals, open communication, learning, and empowering people), each of which measures aspects of patient safety in obstetrics. Nine questions are included in each category, with each question scored on a scale of 1 to 5. The mean response to the nine questions on the 1 to 5 scale is used as the score for each category. The overall score for the CAS is then the mean score on the 1 to 5 scale for the six data elements, and therefore the overall score for the CAS is presented on a scale from 1 to 5 with a maximum score of 5 [21]. The total CAS score as a whole, as well as the scores on the six categories were found to have good internal consistency [21].

The knowledge test and CAS were administered at four time points; pre-MORE^OB^, post-Module 1, post-Module 2, and post-Module 3 (Fig. 1).

#### Sample

As a standard component of participation in the MORE^OB^ program, all MORE^OB^ participants from the 26 hospitals were asked to complete a knowledge test and the CAS at four time points while progressing through the program. In addition to participant scores, the database from Salus Global contained information about job title, hospital site, and a unique identifier which allowed for participant responses to be linked over time. Only those participants who completed the knowledge test and/or CAS at all four time points were included in the final analysis.

#### Data collection

MORE^OB^ participants at the 26 sites completed the standardized knowledge-based assessment tool and CAS at each of the four time points as they progressed through the program. Data were collected by Salus Global on their MORE^OB^ secure on-line platform and released to the research team for analysis.

#### Data analysis

We summarized items from the baseline questionnaire and after each of the three modules using descriptive statistics (means and standard deviations, or frequencies and percentages, as appropriate). Changes in mean scores across the four time points were assessed using linear mixed-effects regression analysis. Repeated measures were accounted for using an unstructured covariance pattern matrix, and clustering at hospital sites was accounted for using a random intercept. Least square means and pairwise comparisons were assessed at the 1% level. All analyses were carried out using SAS v. 9.4.

### Interviews

#### Design

We used a qualitative descriptive design [22, 23]. This design aligned with our evaluation objectives to provide an accurate and comprehensive summary of participants’ perceptions of the effect of the MORE^OB^ program on health care provider knowledge, organizational culture, and barriers and facilitators to implementing and participating in the MORE^OB^ program [22, 24].

#### Materials

A 14-question semi-structured interview guide (Additional file 2) was used to gather in-depth information about interprofessional team experiences with the MORE^OB^ program. The research team reviewed the initial draft interview guide, with modifications made to increase comprehensiveness and clarity (e.g., adding additional probes to elicit information on more concepts, changing wording to make the questions more easily understood). Minor modifications to the guide were made throughout the qualitative data collection period to reflect the timing of the interview in relation to program implementation and to probe new themes emerging from previously conducted interviews.

#### Sample

We used a purposeful sampling approach to recruit interview participants [20]. Specifically, individuals were eligible to participate in an interview if they were (1) health care providers (i.e., nurse, family physician, obstetrician, midwife) and/or administrators/leaders (i.e., nursing or medicine); and (2) participants in the MORE^OB^ program and/or involved in implementing the program as a member of the core team at one of the 26 sites during the two interview recruitment periods (January–April 2015 and April–July 2016).

Permission from each of the 26 hospitals was obtained for Salus Global to release the names and contact information of the MORE^OB^ core team chairs to the research team for the purpose of interview participant recruitment. Interviews were conducted at two time points (post-Module 2 and post-Module 3) to capture the variety of experiences as the MORE^OB^ program progressed. At each of the two time points, an email with an attached recruitment poster was sent by the research team to the MORE^OB^ core team chair at each of the 26 hospitals, with instructions to circulate the invitation to all team members participating in the MORE^OB^ program at their site. Interested participants were instructed to contact the principal investigator to learn more about the study, ascertain eligibility, and schedule a time for an interview. Reminders were sent by the research team to the MORE^OB^ core team chairs to maximize responses. Individuals could participate in interviews at one or both time points. We welcomed participants who volunteered to be interviewed a second time as an opportunity to learn about differences they experienced between modules.

While in qualitative research there are no standardized rules for sample size, we used the concept of information power for guidance [25]. For example, our study aims were relatively narrow (we aimed to explore key dimensions and implementation of the MORE^OB^ program), our purposive sample of participants were specific to our study aims (all had experience participating in and/or implementing the MORE^OB^ program), and we anticipated rich dialogue with participants due to the use of interviewers experienced in both qualitative research and the clinical practice area. These factors, in combination with our experience from past provincial program evaluations, led us to predict that we would need up to 20 participants to provide sufficient information power to answer our research questions.

#### Data collection

Two research staff (one midwife, one nurse) experienced in qualitative interviewing conducted the telephone interviews. Informed written consent was obtained prior to each interview. Interviews lasted an average of 34 min. The first round of interviews (*n* = 9) was conducted between March and April 2015 to learn about participants’ experience with modules 1 and 2 of the MORE^OB^ program. The purpose of conducting the first round of interviews after module 2 was to allow sufficient time for participants to be able to experience and report on any perceived changes to culture and clinical processes in their setting. The second round of interviews (*n* = 10) was conducted between April and July 2016 to learn about participants’ cumulative experiences after completion of module 3 of the MORE^OB^ program and plans for sustainability. Four health care providers were interviewed during both round one and two, meaning that there were a total of 15 unique interviewees. Interviewing, transcription, and analysis proceeded concurrently to permit follow-up of issues emerging from the data, and allow probing of emerging themes in subsequent interviews [22, 23, 26].

#### Data analysis

Digital recordings of the semi-structured interviews were transcribed verbatim. Data were imported into NVivo 11™ [27]. In alignment with our qualitative descriptive study design, we used conventional content analysis to code and analyze our data [22, 28, 29]. Initially, interviews were analysed independently by two study team members. We used an inductive process whereby the codes emerged from the data itself, rather than a pre-existing framework. Specifically, the two study team members independently read each transcript, followed by assigning preliminary codes (i.e., labels that represent the content of words and excerpts in the transcripts [30]). The two coders met every three to five transcripts to compare their coding, reach consensus, and develop and revise the coding scheme. All discrepancies were resolved through discussion between the two coders. As analysis progressed, codes were continually reviewed to identify similarities and differences, and conceptually similar codes were grouped into broader categories [30]. Lastly, we looked for common underlying meaning between categories and created broader themes and sub-themes, which contributed to answering “how” participants experienced the MORE^OB^ program [30]. The analysis of data collected in phase 1 was used to inform revisions to the interview guide for phase 2. For example, questions were added to elicit feedback on perceived differences between modules and on plans for sustainability of the program, and additional probes on key emergent themes such as health care provider confidence were included. Research team members met to discuss the coding scheme, emerging themes and sub-themes, and to build consensus regarding study findings.

## Results

**Fig. 2:**
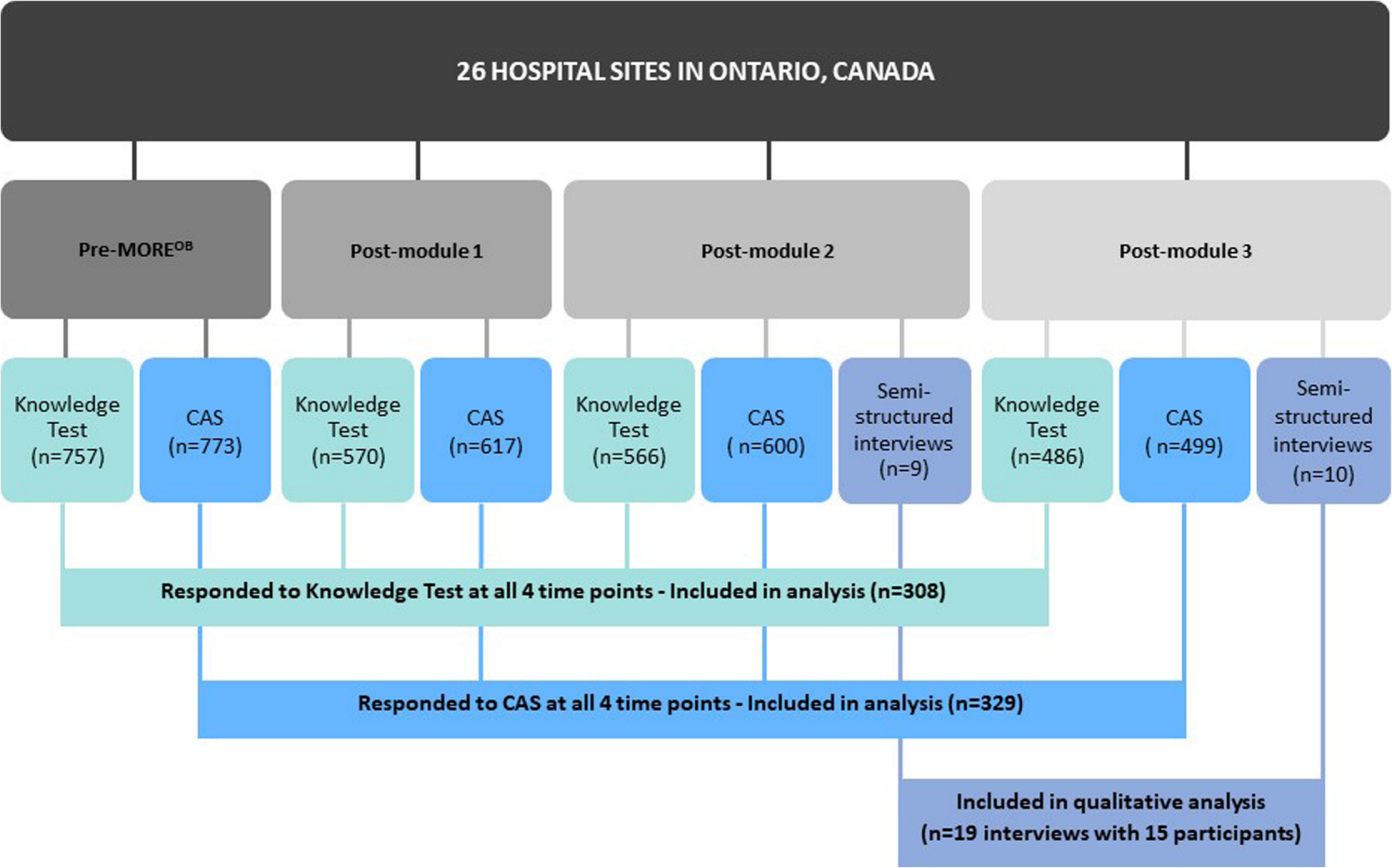
Participant flow diagram

In total, we analyzed data from 308 respondents for the knowledge test, 329 respondents for the CAS, and 19 semi-structured interviews (15 participants) (Fig. 2).

### Surveys

The number of respondents at each time point is shown in Table 2. Of the 962 respondents who completed the knowledge test at any time point, 308 (32%) completed it at all four time points. Of the 988 respondents who completed the CAS at any time point, 329 (33.3%) completed it at all four time points.

**Table 2:** Number of respondents at each time point for the knowledge test and Culture Assessment Survey

Time point	Knowledge Test (*n* = 962) n (%)	Culture Assessment Survey (*n* = 988) n (%)
Baseline	757 (78.7)	773 (77.5)
Post-Module 1	570 (56.3)	617 (62.4)
Post-Module 2	566 (58.8)	600 (60.7)
Post-Module 3	486 (50.5)	499 (50.5)

**Fig. 3:**
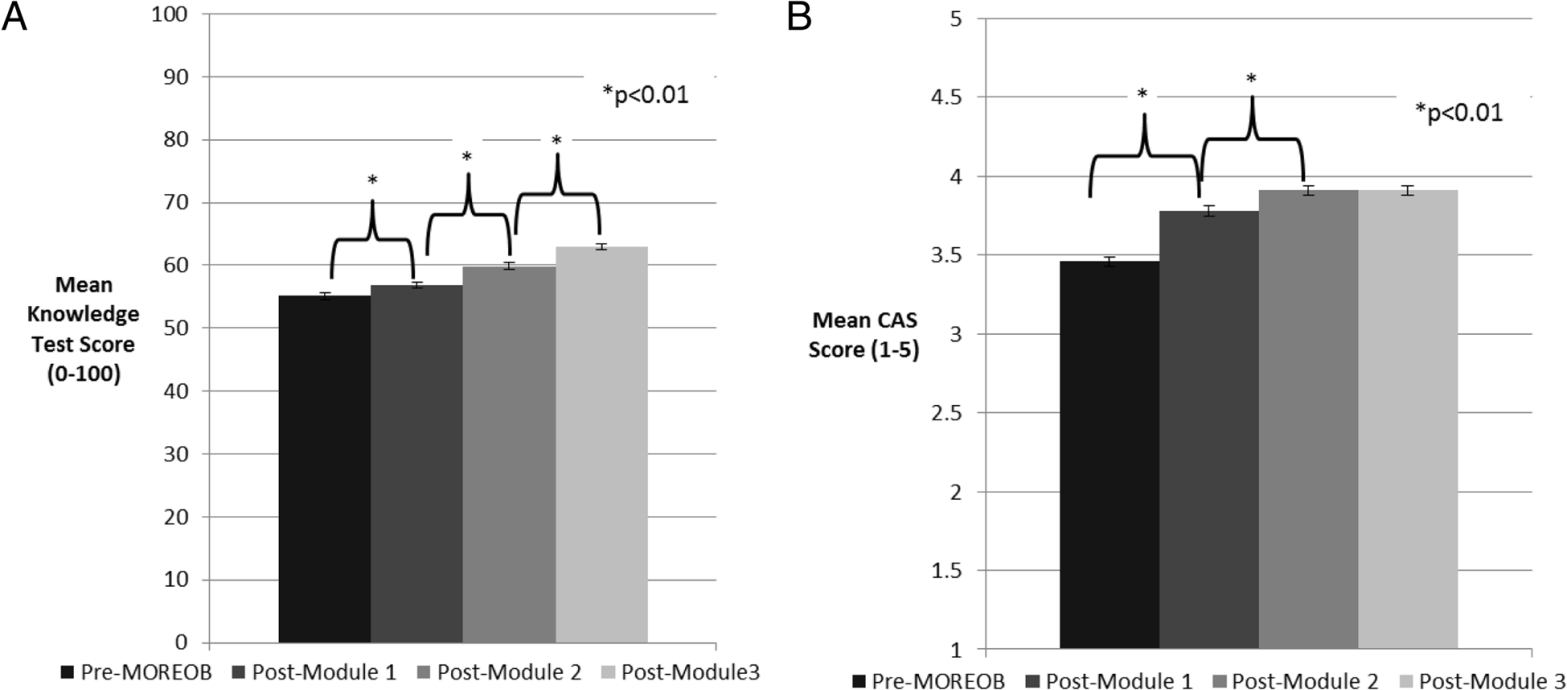
Least square mean estimates and standard errors for Knowledge Tests (**a**), Culture Assessment Survey (**b**). ^*^Pairwise statistical significance at the 1% level. ^**^Differences in Knowledge Test are assessed as mean difference on score from 0 to 100, differences in Culture Assessment Survey scores are assessed as mean differences on score from 1 to 5

Knowledge scores increased by a mean of 7.9 percentage points (95% CI: 7.1 to 8.8) from baseline to post-Module 3 and CAS scores increased by a mean of 0.45 (on a scale of 1 to 5, 95% CI: 0.38 to 0.52). Both increases were statistically significant. Least square mean estimates with standard errors across the four time points are presented in Fig. 3, for the knowledge test and CAS scores.

Increases in knowledge test and CAS scores for each professional group are presented in Table 3 The one respondent in the knowledge test group and two in the CAS group who selected “other” for profession were excluded from this analysis due to low numbers. Increases in both scores were noted for the three professional groups presented, with nurses experiencing the largest increase in knowledge scores, with a mean increase of 9.06 percentage points (95% CI 8.05 to 10.07). Physicians and residents had the greatest increase in CAS scores, with a mean increase of 0.72 on a scale of 1 to 5 (95% CI: 0.57 to 0.88).

**Table 3:** Increases in knowledge test and Culture Assessment Survey scores by professional group, from baseline to post-Module 3^a^

	Knowledge Test (*n* = 307)		Culture Assessment Survey (*n* = 327)	
	n (%)	Mean change^c^ (95% CI)	n (%)	Mean change^d^ (95% CI)
Physicians and residents	47 (15.3)	5.54 (3.91 to 7.16)	52 (15.9)	0.72 (0.57 to 0.88)
Nurses^b^	233 (75.9)	9.06 (8.05 to 10.07)	243 (74.3)	0.37 (0.29 to 0.45)
Midwives	27 (8.8)	2.59 (0.62 to 4.55)	32 (9.7)	0.59 (0.37 to 0.82)

*CI*, Confidence Interval

^a^For participants with responses at four time points

^b^One Registered Practical Nurse

^c^Reflects mean difference on score from 0 to 100

^d^Reflects mean difference on score from 1 to 5

### Interviews

Between March 2015 and July 2016, 19 telephone interviews were conducted with a total of 15 health care providers from 11 sites (Table 4).

**Table 4:** Qualitative interview participant demographics

	N (%)
Gender	
Female	14 (93.3)
Male	1 (6.7)
Age	
35–44	6 (40.0)
45–54	8 (53.3)
55–64	1 (6.7)
Current professional practice	
Registered nurse	6 (40.0)
Family physician	5 (33.3)
Registered midwife	2 (13.3)
Obstetrician	1 (6.7)
Administrator	1 (6.7)
Number of years involved with intrapartum care	
0–4	1 (6.7)
5–9	4 (26.7)
10–14	2 (13.3)
15–19	2 (13.3)
20–24	1 (6.7)
25–29	4 (26.7)
30–34	1 (6.7)
Member of MORE^OB^ core team	
Yes	11 (73.3)
No	4 (26.7)

*MORE*^*OB*^, Managing Obstetrical Risk Efficiently

**Fig. 4:**
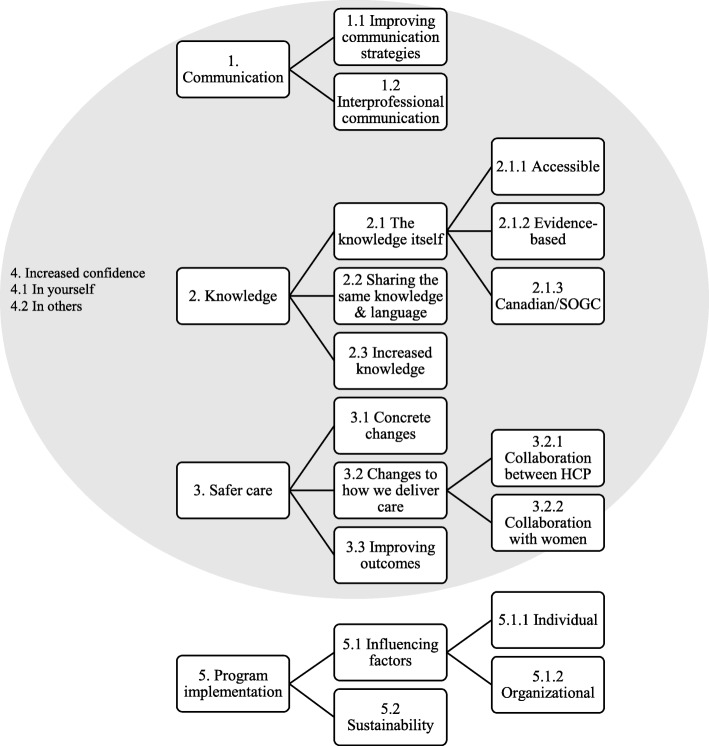
Organization of themes and sub-themes

Overall, participants reported being satisfied with the MORE^OB^ program. Five main themes and their respective sub-themes were identified: communication, knowledge, safer care, increased confidence, and program implementation (Fig. 4). Table 5 provides participant quotes to support our findings [29].

**Table 5:** Themes and sub-themes with example quotes

Theme	Sub-theme		Example Quote
1. Communication	1.1 Improving communication strategies		It’s having access to the practice standards that are all printed in the MORE^OB^, like all the clinical knowledge and then learning some of the communication pieces, like the Take Five and learning to debrief after even a normal delivery and using like the SBAR and things, so utilizing that in practice. (Interview 1A – Registered Nurse, Post-Module 2)
	1.2 Interprofessional communication		There’s been increased engagement amongst and between the groups participating, so midwives, physicians and nurses. So we’re certainly talking about stuff together. (Interview 3A - Family Physician, Post-Module 2)
2. Knowledge	2.1 The knowledge itself	2.1.1 Accessible	I have really liked the availability to go on to see the modules online, you know, at the drop of a hat. We have electronic medical record at our hospital so there’s computers all over the place. So it’s quick. It’s easy to access things. (Interview 5A – Family Physician, Post-Module 2)
		2.1.2 Evidence-based	Instead of my searching for all the guidelines and going into ACOG and SOCG, it’s [MORE^OB^] kind of one-stop shopping, right? So then it’s kind of like I always say ‘yes, these are guidelines and our clinical judgement comes into play, but this is kind of what MORE^OB^ summarizes,’ which is always up-to-date because they’re looking at it from a yearly basis and they’re collectively gathering the data so I don’t have to, so it saves me a ton of time that way. (Interview 1B – Registered Nurse, Post-Module 3)
		2.1.3 Canadian/SOGC	So because the information was very readable, because it’s Canadian based with SOGC back-up and I’ve done the ALARM course a few times so it’s basically the same content. So it’s something we feel we can buy into. It’s something we feel like this is the norm. This is the expectation. This has been researched. This makes sense. (Interview 3A – Family Physician, Post-Module 2)
	2.2 Sharing the same knowledge and language		So we have some content resources and some communication resources to refer to and so when they’re talking to me about something, then I know that we’re talking about the same thing. I know what they’re saying and that what I’m saying to them is sort of being understood on the same level. (Interview 14A – Obstetrician, Post-Module 3)
	2.3 Increased knowledge		For us, our education program for our labour and delivery nurses were they got six shifts in a labour … they didn’t even have to see a delivery to be an OB nurse … So that was kind of the education level when I started here and of course, because we only do 100 deliveries a year, it’s hard for them to get enough volume. So in a centre like ours, I think you can really see the impact of a program like this. (Interview 9A - Family Physician, Post-Module 2)
3. Safer care	3.1 Concrete changes		Previously there were a number of pieces of equipment we didn’t have in the room so we’ve acquired equipment, just there was a lot of that happening actually where it’s like ‘oh my gosh, like we don’t even have these things’ … I think probably in some cases people recognized issues in the past but didn’t have a way of communicating those concerns so I think frequently there was nothing to sort of back them up and show why this should be changed. And now with MORE^OB^ it gave the research and the explanation of here are the things you should try to implement in your site. (Interview 4B – Registered Midwife, Post-Module 3)
	3.2 Changes to how we deliver care	3.2.1 Collaboration between HCP	I really believe that because of the MORE^OB^ principles of teamwork and openness and transparency and the debriefing methods that the safety for the actual client has improved markedly. (Interview 6A - Registered Nurse, Post-Module 2)
		3.2.2 Collaboration with women	There’s much more strengthened education that nurses are sharing with patients, helping them to better understand even when things are going in a difficult direction for a patient, helping them and their partners to better understand what’s unfolding and what to expect. (Interview 7B – Administrator, Post-Module 3)
	3.3 Improving outcomes		We had a post-partum hemorrhage the other day and how it was handled, again because of those modules and knowing the steps to perform, they were done quickly and that situation was dealt with. And again, the mom was okay and the baby was okay after the crisis had occurred. (Interview 11A - Registered Nurse, Post-Module 3)
4. Increased confidence	4.1 In yourself		It’s more confidence and being empowered. Doing the MORE^OB^ program empowers us to have knowledge first of all, but also gives us ways to communicate to each other and just mobilizes it. It just makes you feel empowered to speak your mind. And no questions or no comments are wrong, that it’s an opportunity to learn. (Interview 12A - Registered Nurse, Post-Module 3)
	4.2 In others		But I think what it [MORE^OB^] does for their group is I think it begins to increase not only their confidence in their co-workers but their expectations of their co-workers … they know that this knowledge is available at present and expectation and can have confidence that their team, even if they’re not really experienced team members, are expected to know this. (Interview 10A - Registered Nurse, Post-Module 3)
5. Program implementation	5.1 Influencing factors	5.1.1 Individual	My nursing staff in particular have been incredibly hungry around new knowledge and this is really, the timing of this program has been perfect for them and we don’t have a dedicated nursing educator here in our small hospital and so they’ve really, really, really expressed incredible gratitude in terms of being able to participate in this program. (Interview 7A – Administrator, Post-Module 2)
		5.1.2 Organizational	I mean I think we all had pretty high hopes that this was going to sort out some issues and problems. And I think one of the biggest learnings for me is that the best education program in the world can’t solve a culture issue if you don’t have leadership and you don’t have people walking the talk and really bringing home the importance of those issues, it’s not going to stick or people aren’t going to really adopt the behaviour change and people revert back to their old ways pretty quickly. (Interview 13A - Family Physician, Post-Module 3)
	5.2 Sustainability		You can’t do a program for three years and they rub their hands and say we’re done now. There has to be ongoing passing the talent on in the correct way. This is what the basis of our unit is and it should be across the whole organization. (Interview 12A - Registered Nurse, Post-Module 3)

*ACOG* The American College of Obstetricians and Gynecologists, *ALARM* Advances in Labour and Risk Management, *MORE*^*OB*^ Managing Obstetrical Risk Efficiently, *SBAR* Situation, Background, Assessment, Recommendation, *SOGC* The Society of Obstetricians and Gynaecologists of Canada

#### Theme 1: communication

An important focus of the MORE^OB^ program was *improving communication strategies*. Participants identified specific communication strategies learned through the MORE^OB^ program implemented in practice, including ‘Take Five’ and ‘SBAR’ (Situation, Background, Assessment, Recommendation), which were implemented during debriefing about cases.

Participants perceived increased *interprofessional communication,* resulting from bringing the different professions together to participate in the program, sometimes for the first time. These opportunities for interprofessional communication and teamwork led to participants perceiving a more familiar and collegial work environment.

#### Theme 2: knowledge

Participants valued the *knowledge itself* gained from the MORE^OB^ program. MORE^OB^ was described as a comprehensive, time efficient, and accessible resource as all information is contained in one place, both in-print and online. In addition, participants perceived that the content of MORE^OB^ had been well-researched, was evidence-based, and relevant to the local context.

Participants described how participating in the MORE^OB^ program allowed all team members to *share the same knowledge and language,* put everyone “on the same page,” and gave everyone a common reference point.

All participants described how MORE^OB^ *increased their knowledge* base and that of their colleagues. For some, this increase in knowledge was a reminder of the importance of ongoing training to maintain current knowledge and skills. The MORE^OB^ program was described as being particularly helpful in a small hospital setting with low birth volumes where it can be difficult for new obstetrical nurses to gain sufficient experience in obstetrics.

#### Theme 3: safer care

Participation in MORE^OB^ resulted in the perception that the health care team was providing safer care to patients. Participants identified *concrete changes* made on their units due to their participation in MORE^OB^. Examples of concrete changes included development or updating of policies and procedures, creating disaster carts with appropriate equipment, changes to clinical practices, posting algorithms on walls for clinicians to view, conducting case audits, and utilizing debriefing methods.

Participants described how MORE^OB^ *changed the way they delivered care*. First, participants described improved collaboration with their colleagues, which resulted in better ‘team work, openness, and transparency’, which ultimately improves safety for patients. For those sites who described pre-existing hierarchical relationships between obstetrical care providers, participants described a decrease in hierarchy within their team. However, a couple of sites described still struggling with hierarchy and were still working to change the culture at their sites. In addition, participants identified that participation in the MORE^OB^ program improved the patients’ experience by improving their collaboration with women and emphasizing the importance of patient centred care.

Generally, participants could not speak definitively as to whether their hospital’s participation in the MORE^OB^ program had led to *improved outcomes* such as decreased maternal and infant morbidity and mortality, as they did not have data to refer to during the interview. One site had these data and stated they observed a reduction in critical events since participating in MORE^OB^. Overall, participants believed that the increased knowledge and concrete changes were ultimately improving care, outcomes, and patient experience.

#### Theme 4: increased confidence

Participants described the impact MORE^OB^ had on their *self-confidence*, particularly nurses who were described as being empowered through the MORE^OB^ program by increasing their knowledge and skills and improving their team communication. In addition, participants described an *increase in their confidence* in the knowledge, skills, and judgments of their team members. As MORE^OB^ participants improved communication, increased their knowledge base, and improved their ability to provide safer care, their confidence in themselves and others increased.

#### Theme 5: program implementation

Participants described different strategies used for implementing the MORE^OB^ program, including use of huddle boards, staff emails and staff newsletters. In general, participants described following the MORE^OB^ activities as intended, with sites modifying the activities to make it more relevant for their setting or to fit it into limited time frames as needed. Some participants stated that the MORE^OB^ program plan was not clear, even after attending the orientation, and felt that additional detail on upcoming activities would be beneficial.

Participants described several *influencing factors* that affected how easy or difficult it was to implement the MORE^OB^ program in their context. For example, buy-in was increased when team members were interested in the content of the module and “hungry for new knowledge.” Participants also identified organizational-level barriers including limited time of staff and lack of funding to pay staff for their time to participate in the program, and a baseline organizational culture that was more resistant to change. Organizational-level facilitators included engaged hospital administration and the presence of MORE^OB^ champions who were highly committed to the implementation of the program.

Participants described varying levels of awareness regarding the *sustainability* of the MORE^OB^ program at their hospital once their three-year funding ended. Participants were keen to keep MORE^OB^ active at their hospital, and many sites were pursuing continued involvement, either formally with Salus Global, or when funding was not available, through internally-led education programs and skills drills. Regardless of the specific strategy, sustainability of the knowledge and skills gained through MORE^OB^ was identified as a priority.

Staff turnover and new hospital priorities were identified as ongoing challenges to sustainability of the program. As new people joined the unit, they had not participated in the initial MORE^OB^ program modules with the remainder of the team which presented challenges as the team was “storming and forming” all over again. A key challenge for sustainability of the program was cost, with participants perceiving that the program should be kept at a reasonable price to ensure institutions and providers can benefit from it.

## Discussion

Our mixed-methods study evaluated the effect of the MORE^OB^ program on participant knowledge, perception of organizational culture, and experiences implementing and participating in the program. Findings from both the quantitative and qualitative components of this study demonstrated increases in knowledge and positive perceptions of organizational culture. Findings from the qualitative component revealed that participants spoke positively of the program and the benefits of participating. In addition, barriers and facilitators to implementation and sustainability were identified.

### Participant knowledge

The increases in knowledge scores occurring for all professional groups resonate with findings from our qualitative analysis, with participants describing MORE^OB^ benefits such as increased knowledge and sharing a common knowledge, as well as increased confidence in themselves and in their teammates as a result of this increased knowledge.

Data collected in a previous study revealed similar results on the knowledge tests in Canadian hospitals after MORE^OB^ implementation, with all professional groups showing an increase in knowledge from baseline to post-Module 3 [18]. As in our evaluation, nurses were the professional group with the largest increase in knowledge test scores from baseline to post-Module 3 [18]. This may be partially explained by the small size of the hospitals included in our sample, with approximately 80% providing level 1 (low-risk) care and approximately 70% having a birth volume of less than 500 per year. At such small sites, many nurses practice in multiple areas (e.g., medical/surgical) and are cross-trained to provide obstetrical care as needed. Conversely, physicians and midwives practicing at these sites specialize in obstetrics and thus may have had a greater depth of baseline knowledge in this area.

### Participant perception of organizational culture

Our study showed an overall significant increase in the safety culture from baseline to post-Module 3, with scores increasing almost half a point out of 5 from pre-MORE^OB^ levels. Likewise, in the previous Canadian evaluation, there was a significant increase on all six elements of the CAS [18]. Our qualitative findings were aligned with these results, with participants giving examples of improvements related to the safety culture in their obstetric units, including strengthened teamwork and communication.

Team work, which is one of six categories on the CAS, has been identified as an essential element for reducing maternal and infant morbidity and mortality, as well as improving patient experience in obstetrics [31]. Characteristics of team training to improve obstetrical outcomes include use of in-house rehearsals and debriefing, interprofessional training, and access to and training on evidence-based clinical guidelines [31], all of which are consistent with features of the MORE^OB^ program and may have facilitated the positive culture changes we saw in our evaluation.

From the qualitative data, we learned that despite participation in the MORE^OB^ program, some sites still struggled to overcome issues with hierarchy on the interdisciplinary team, which is important as it can prevent health care providers from speaking up [32]. The ongoing challenges with hierarchy we observed in our interviews may not be surprising, given that hierarchal gradients continue to exist in many health care settings, despite attempts to reduce these hierarchies within and between professions [33]. One industry that has successfully implemented interventions to reduce hierarchy between team members and improve culture and safety is aviation [33]. Specifically, the use of Crew Resource Management (CRM) training helps to flatten the hierarchy, minimize power differentials, and improve communication by training all crewmembers, regardless of role, seniority, or experience, to speak up with safety concerns [33, 34]. Parallels have been drawn between aviation and healthcare related to safety management [35, 36] and the application of CRM training in health care was recommended in the 1999 To Err is Human Report [5]. Examples of CRM in the field of obstetrics specifically can be found in the literature [7, 37–40]. While the MORE^OB^ program uses HRO principles [12] applied in the aviation industry, additional strategies and ongoing training opportunities beyond the 3-year MORE^OB^ program may be required for sites to continue to decrease interprofessional hierarchy and improve their ability to work and communicate as a team.

The varying experiences in culture change found in our qualitative interviews speak to the importance of the context in which the MORE^OB^ program is being implemented. The influence of site context on culture change has been observed in other healthcare settings, with a longitudinal mixed-methods study of 10 hospitals participating in a quality collaborative intervention reporting distinct differences between the hospitals with substantial culture change versus those without. Hospitals with positive culture change employed an interprofessional and inter-hierarchal approach, reported high levels of participation, and utilized strategies for managing conflict and maintaining engagement [41]. Although the MORE^OB^ program aims to improve the safety culture in birthing units, the extent to which this can be improved may be influenced by specific hospital and team characteristics.

While our quantitative and qualitative findings support the effectiveness of the MORE^OB^ program in improving several dimensions of a culture of safety, it is important to acknowledge that other patient safety initiatives in obstetrics, such as CRM team training, the Comprehensive Unit-based Safety Program (CUSP), and other locally developed quality improvement initiatives, have also demonstrated improvements in health care provider safety attitudes [37, 38, 42, 43]. Examples of key elements of these other initiatives included implementing an obstetric patient safety nurse [37, 42] and an obstetrics patient safety committee [37, 42, 43], using interdisciplinary team training [37, 38, 42, 43], and implementing improved communication systems and strategies [37, 38, 42, 43]. All reported improvements in safety culture after implementation of their respective patient safety initiatives, with significant improvements in teamwork culture [37, 42], safety culture [37, 42], job satisfaction [42, 43], working conditions [43], and perceptions of management [37, 42, 43].

Lastly, there is evidence to suggest that positive workplace and organizational culture is associated with improved patient outcomes including decreased mortality rates, adverse events, readmission rates, and increased patient satisfaction across a variety of health care settings [44]. While our study found a significant increase in the CAS which was largely supported by our qualitative findings, it remains unknown how this positive change in culture translates to clinical outcomes in our setting.

### Barriers and facilitators to implementing and participating in MORE^OB^ program

Overall, interview participants expressed satisfaction with their participation in the MORE^OB^ program. Several individual- and organizational-level facilitators and barriers to implementation and maintenance of the MORE^OB^ program were identified, some of which are consistent with factors reported in the literature, and align with existing theoretical frameworks [45]. For example, participants indicated that *social influences* [45] such as the effect of the core team and champions, influenced their team’s success implementing the MORE^OB^ program. This was similar to lessons learned in another Canadian evaluation, where the hospitals’ MORE^OB^ core team was identified as essential to driving the participation of members [18]. Second, our participants described the effect of the *environmental context and resources* [45] such as the availability of funding to support the MORE^OB^ program and baseline organizational culture. With limited financial, physical, and human resources within health care organizations, running and sustaining the MORE^OB^ program can present a challenge.

Previous research suggests potential strategies to facilitate implementation and uptake of the MORE^OB^ program include increased leadership engagement, conducting a baseline barriers assessment, and use of ongoing monitoring and evaluation at a local level. First, leadership has been identified as critical for developing a culture of safety [46], and in the case of MORE^OB^, the support of senior administration is key to successful implementation of the program, through direct participation on the core team, and through supporting their work [18]. Next, an assessment prior to MORE^OB^ program implementation may assist hospitals to identify individual- and organization-level barriers, and identify existing strengths and resources that may be used to facilitate implementation [47]. Lastly, participants in our study were largely unable to definitively state whether their team’s participation in the MORE^OB^ program had translated to improved patient outcomes at their hospital. Evaluating key outcomes using local data is an important component of the knowledge translation process [47] to improve patient safety. Ongoing monitoring and evaluation of hospital-based patient safety outcomes may assist with keeping teams engaged with the MORE^OB^ program by showing participants where they are (or are not) making a difference, and identifying areas of improvement for the MORE^OB^ core team related to program implementation and participation.

### Implications

While our evaluation showed positive findings from the participant surveys and qualitative interviews, understanding the actual impact on perinatal outcomes is important, because a patient safety program should really translate into improved outcomes. As part of the larger evaluation we reported that these findings did not translate into a statistically significant reduction in adverse maternal and neonatal outcomes at the provincial level [17]. Despite the lack of reduction in adverse outcomes, it seems clear from the results we present in this paper that the MORE^OB^ program increased knowledge, changed the patient safety culture, and improved the work environment at many participating hospitals. For example, participants in the interview component of our study identified other improvements that were not within the scope of this current study to assess quantitatively. Specifically, interview participants spoke of perceived improvements from the MORE^OB^ program related to patient experience, health care provider confidence, and interprofessional communication. These additional benefits are important to individuals and health systems because: (1) improved patient experience is an important goal for health care systems [48], and (2) health care provider confidence and communication are important individual-level factors influencing “speaking up behavior” [49], an essential component of a patient safety culture [50]. Given the potential importance of these additional health care provider and patient outcomes, future work to comprehensively assess the effect of the MORE^OB^ program on these constructs using validated measures may be warranted.

Considering the overall positive results from the surveys and interviews, it is not surprising that most participants expressed a desire to keep the program active at their centers. Future work to create a program model that is affordable and sustainable for participating hospitals is needed. Since the completion of this evaluation, Salus Global has launched MORE^OB^ 2.0 and MORE^EX^, which can support health care teams in acute settings beyond obstetrics [51]. Future evaluations of the revised MORE^OB^ 2.0 program may consider our lessons learned from the Alberta [15] and Ontario provincial evaluations as well as the Canadian evaluation [18], and eventually provide comparative data.

### Strengths and limitations

The use of a mixed-methods approach is an important strength of this study. We used data from two surveys, carried out at four time points as participants progressed through the MORE^OB^ program. As well, we included data from qualitative interviews carried out with 15 participants, including an administrator, nurses, midwives, and physicians, providing rich in-depth information on the MORE^OB^ program. The fact that findings from both methods were convergent regarding improvements in knowledge and safety culture supports the effect of the program on these outcomes.

To our knowledge, ours is only the second paper to publish results of the knowledge test and CAS at baseline and after implementation of the three MORE^OB^ modules. Our findings are similar to those published previously, and are an important addition to the literature due to the limited methodologic and sampling information provided in the earlier publication [18]. Further, we have used a more robust analytic approach for analyzing repeated measures survey data.

A limitation is the inclusion of only those 26 hospitals who received provincial funding to implement the MORE^OB^ program in 2013. These sites likely differ from the 67 hospitals that voluntarily initiated participation in the MORE^OB^ program prior to 2013, independently of any funding. As well, for the knowledge test and CAS, all of the respondents were participants in the MORE^OB^ program, meaning that we do not have data on non-participants. This is problematic, in that one would expect knowledge scores to increase over a four-year time period, with increased time in the workplace. Due to this limitation, we cannot attribute the increases we observed solely to participation in the MORE^OB^ program. Additionally, the majority of respondents did not complete the CAS and knowledge test at all four time points. This is not surprising given staff turnover rates on obstetric units, however, our analyses were therefore limited to the one third of respondents who completed all of the assessments. Furthermore, it is difficult to know the clinical effect of the increases in knowledge and culture that were observed. Another limitation is the lack of data available on survey respondents, for example, we do not know their age or number of years in the workforce, which could influence survey scores. While our qualitative interviews included participants from diverse hospitals and professional backgrounds, only one obstetrician participated in an interview.

Lastly, it is important to acknowledge that our sample of 15 unique interviewees only included individuals from 11 out of the 26 potential hospitals. While efforts to maximize recruitment were made by communicating through the chair of the MORE^OB^ core team at each site, by sending multiple email reminders, and by accommodating all participant schedules for interviews, we still only managed to capture participants from about 40% of eligible hospitals. Nonetheless, the information we obtained from these interviews provided valuable insight into the MORE^OB^ program and its implementation to answer our research questions, although these findings may not be transferable to individuals and teams at the remaining sites.

## Conclusion

Results of this mixed-methods study suggest that participants were satisfied with their participation in the MORE^OB^ program. Barriers and facilitators to implementation and sustainability of the MORE^OB^ program were identified. Strategies to address barriers, maximize facilitators, and enhance sustainability of the MORE^OB^ program are needed. Our results also suggest that the program may have a positive effect on participant knowledge and organizational safety culture. Further work to understand how these improvements translate to outcomes for patients and health care providers is needed.

## Additional files

Additional file 1: MORE^OB^ program goals. This file provides the seven main goals of the MORE^OB^ program we evaluated in this study. (DOCX 15 kb)

Additional file 2: Semi-structured interview guide. This file provides the 14 questions that were used during the semi-structured interviews. (DOCX 14 kb)

## Data Availability

The qualitative data analyzed during this study (i.e., interview transcripts) are not publicly available due to them containing information that could compromise research participant privacy/consent. The data that support the findings from the survey component of this study are available from Salus Global but restrictions apply to the availability of these data, which were used under license for the current study, and so are not publicly available. Data are only available with permission from Salus Global and the participating hospitals.

## Abbreviations

ACOG: The American College of Obstetricians and Gynecologists
ALARM: Advances in Labour and Risk Management
CAS: Culture Assessment Survey
CRM: Crew Resource Management
HIROC: Healthcare Insurance Reciprocal of Canada
HRO: High Reliability Organization
MOHTC: Ministry of Health and Long-Term Care
MORE^OB^: Managing Obstetrical Risk Efficiently
SBAR: Situation, Background, Assessment, Recommendation
SOGC: Society of Obstetricians and Gynaecologists of Canada
