# Supplementary material for: A mixed-methods evaluation of the MOREOB program in Ontario hospitals: participant knowledge, organizational culture, and experiences
Source: BMC Health Serv Res. 2019 Jul 8;19:460. doi: 10.1186/s12913-019-4224-9 (PMC6615285; doi:10.1186/s12913-019-4224-9)
Supplement: Supplementary file 1 — MOREOB program goals. This file provides the seven main goals of the MOREOB program we evaluated in this study. (DOCX 15 kb) [file 12913_2019_4224_MOESM1_ESM.docx]

| **Additional File 1. MORE^OB^ program goals** [14] |
| --- |
| - Maintain and apply an evidence-based body of knowledge - Perform fundamental skills confidently and automatically - Manage emergencies in a standardized and well-coordinated fashion - Communicate and work effectively as a collaborative, interprofessional community of practice in partnership with the patient and the family to promote safe care - Use interprofessional reflective learning to evaluate processes and outcomes of clinical practice and organizational systems - Be vigilant, and anticipate potential safety risks - Modify care practices and organizational systems to reduce safety risks and prevent harm |
