# Supplementary material for: A mixed-methods evaluation of the MOREOB program in Ontario hospitals: participant knowledge, organizational culture, and experiences
Source: BMC Health Serv Res. 2019 Jul 8;19:460. doi: 10.1186/s12913-019-4224-9 (PMC6615285; doi:10.1186/s12913-019-4224-9)
Supplement: Supplementary file 2 — Semi-structured interview guide. This file provides the 14 questions that were used during the semi-structured interviews. (DOCX 14 kb) [file 12913_2019_4224_MOESM2_ESM.docx]

**Additional file 2.** Semi-structured interview guide

|  | **Interview Question** |
| --- | --- |
| 1 | What (if anything) has changed with the implementation of the MORE^OB^ program in your hospital? |
| 2 | What are some of the enablers experienced by health care providers in your institution for implementation of the patient safety activities associated with the MORE^OB^ program? |
| 3 | What are some of the barriers experienced by health care providers in your institution for implementation of the patient safety activities associated with the MORE^OB^ program? |
| 4 | Tell me about the process your hospital used for implementing the MORE^OB^ activities. |
| 5 | How has the implementation of MORE^OB^ changed your practice? |
| 6 | How has the implementation of MORE^OB^ changed the way the team practices? |
| 7 | We heard in previous interviews that for many health care providers MORE^OB^ increases their confidence. Would you say this is true for you? How so? |
| 8 | In your opinion, does the availability of the MORE^OB^ program in your setting improve care for women? |
| 9 | Do you have any examples of how the availability of the MORE^OB^ program in your setting has improved patient outcomes? |
| 10 | In your opinion, does the availability of the MORE^OB^ program in your setting improve the working environment for care providers? |
| 11 | Have you seen any concrete changes in your setting as a result of involvement with the MORE^OB^ program? |
| 12 | How does your hospital plan on sustaining the MORE^OB^ program? |
| 13 | If you are a member of the Core Team in your institution responsible for the implementation of the program, what has been your greatest success to date? What has been your greatest challenge to-date? |
| 14 | Is there anything else you want to tell us about the MORE^OB^ program? |

*MORE^OB^, Managing Obstetrical Risk Efficiently*
